# Gonadal steroids as predictors of sex differences in tuberculosis outcomes

**DOI:** 10.1172/jci.insight.200877

**Published:** 2026-06-16

**Authors:** Djeneba Dabitao, Bocar Baya, Ibrahim Sanogo, Amadou Somboro, Mamadou Wague, Mamadou D. Coulibaly, Isaac Koloma, Mahamadou Kone, Mohamed Nantoume, Nadie Coulibaly, Stephane Behinan, Mariam Coulibaly, Mamadou Perou, Moumine Sanogo, Ayouba Diarra, Seydou Samake, Bassirou Diarra, Mahamadou Diakite, Souleymane Diallo, Yacouba Toloba, Chad Achenbach, Jane L. Holl, Seydou Doumbia, Robert Murphy, William Bishai, Sabra Klein

**Affiliations:** 1 University Clinical Research Center, Faculty of Pharmacy and Faculty of Medicine and Odonto-Stomatology, University of Sciences, Techniques, and Technologies of Bamako, Bamako, Mali.; 2 Department of Pneumophtisiology, Teaching Hospital of Point-G, Bamako, Mali.; 3 Division of Infectious Diseases and Havey Institute for Global Health, Feinberg School of Medicine, Northwestern University, Chicago, Illinois, USA.; 4 Biological Sciences Division, University of Chicago, Chicago, Illinois, USA.; 5 Department of Infectious Diseases, Center for Tuberculosis Research, Johns Hopkins School of Medicine, Baltimore, Maryland, USA.; 6 W. Harry Feinstone Department of Molecular Microbiology and Immunology, Johns Hopkins Bloomberg School of Public Health, Baltimore, Maryland, USA.

**Keywords:** Immunology, Infectious disease, Cytokines, Sex hormones, Tuberculosis

## Abstract

**BACKGROUND:**

Recent evidence suggests a role for biological factors in increased risk for active pulmonary tuberculosis (PTB) among males. We determined the relationship between alterations in gonadal steroids, tuberculosis (TB) disease status, and treatment outcomes.

**METHODS:**

We conducted a prospective cohort study in Mali of treatment naïve males and females with laboratory-confirmed PTB, latent TB infection (LTBI), and healthy controls of similar ages.

**RESULTS:**

Prior to treatment, males with PTB had lower testosterone concentrations compared to males with LTBI or healthy males. Reduced testosterone concentrations in males with PTB were transient, returning to healthy ranges by month 2 of treatment, which corresponded to the end of intensive TB treatment. Estradiol concentrations in females were not altered by PTB or infection status yet increased at month 6 of treatment. Testosterone, but not estradiol, was a strong predictor of cure during treatment. Testosterone, but not estradiol, concentrations in PTB cases were inversely correlated with serum IFN-γ, IL-6, and IL-2. Concentrations of IL-17 and IL-10 were lower in males than females at the end of TB treatment.

**CONCLUSION:**

Our results suggest that TB-induced changes in testosterone concentrations during PTB and in response to treatment occur in males and could contribute to sex differences in TB pathogenesis.

**FUNDING:**

The Fogarty International Center and the Office of Research on Women’s Health (K43TW011426); the Institute for Global Health at the Feinberg School of Medicine of the Northwestern University (Catalyzer Award); 2021 TWAS Abdool Karim Award; NIH grants R37AI167750 and 5D43TW010350.

## Abstract 2

We found that changes in the concentrations of testosterone, a dominant male hormone, is an independent predictor of treatment outcome in males with tuberculosis. Our findings may be useful in monitoring treatment outcomes in addition to traditional microbiological and clinical assessment.

## Introduction

Tuberculosis (TB) is an ancient disease that continues to cause high morbidity and mortality globally, despite the availability of preventive measures and effective treatments. It is estimated that 23% of the world population is infected with bacterial species of *M. tuberculosis* complex (MTBC) group (1), among whom 5%–10% will develop active pulmonary TB (PTB) later during their lifetime (2, 3). Risk factors associated with susceptibility to PTB include HIV infection, malnutrition, diabetes, smoking, alcohol abuse, use of immunosuppressive drugs, poverty, overcrowding, silicosis, and male sex (4, 5). Male sex, in particular, is an emerging and underexplored risk factor identified in various epidemiological observations. Notably, the global burden of PTB is characterized by an excess of TB disease in adult males compared with adult females despite notable gaps in case detection rates and notification between males and females (6). In 2023, 55% of incident TB occurred in men compared with only 33% in women (7). Sex differences in TB incidence also are observed in age-standardized mortality rates, which were found to be 2 times higher in males than in females (8). Likewise, we and others have found that male sex is associated with low smear conversion rates during the intensive phase of treatment (9, 10), and higher risk of mortality 9 months after the start of treatment (11).

Despite these compelling epidemiological data, the mechanisms driving associations between male sex and PTB have not been fully elucidated. Traditionally, non-biological factors, such as smoking, alcohol use, health-seeking behaviors, and poor treatment adherence, which are also known to be influenced by gender, were considered to be the main drivers of increased TB burden and worse treatment outcomes for men (12). However, a growing body of evidence also suggests a role for biological factors such as gonadal steroids (i.e., testosterone in males and estradiol in females). Evidence of a potential role of gonadal steroids in the association between PTB and male sex stems from a seminal observation in eunuch (i.e., castrated) men, who were less likely to die of PTB than non-castrated men (13), suggesting a potential role of androgens in promoting unfavorable TB outcomes in males. Based on global TB reports, TB incidence is not increased in males until after puberty, indicating an association between changes in gonadal steroids and differential susceptibility to TB disease (7, 14). Laboratory studies further reveal that gonadally intact male mice are more likely than females to die from mycobacterial infection, and that male-biased death is reversed by castration, further supporting a potential role of androgens in TB pathogenesis (15, 16). Accordingly, higher mycobacterial load, heightened levels of inflammatory cytokines, and impaired B cell follicle formation were found in the lungs of male compared with female mice (15, 17, 18). Therefore, both human and animal studies suggest that gonadal steroids have a role in promoting increased PTB incidence and worse outcomes among males.

In this longitudinal, prospective, cohort study, we assessed the relationship of gonadal steroids, specifically, total testosterone in males and total estradiol in females, with 2 clinical forms of TB (i.e., active and latent). We compared concentrations of gonadal steroids in sex- and age-matched male and female patients with active or latent TB infection with comparison to healthy sex- and age-matched controls. We determined whether changes in gonadal steroids were associated with PTB treatment outcomes. Lastly, to understand the influence of gonadal steroids on sex differences in TB disease outcomes, we examined longitudinal changes in inflammatory cytokine levels during treatment for PTB and their correlation with gonadal steroids in males and females.

## Results

### Recruitment of study participants.

**Figure 1:**
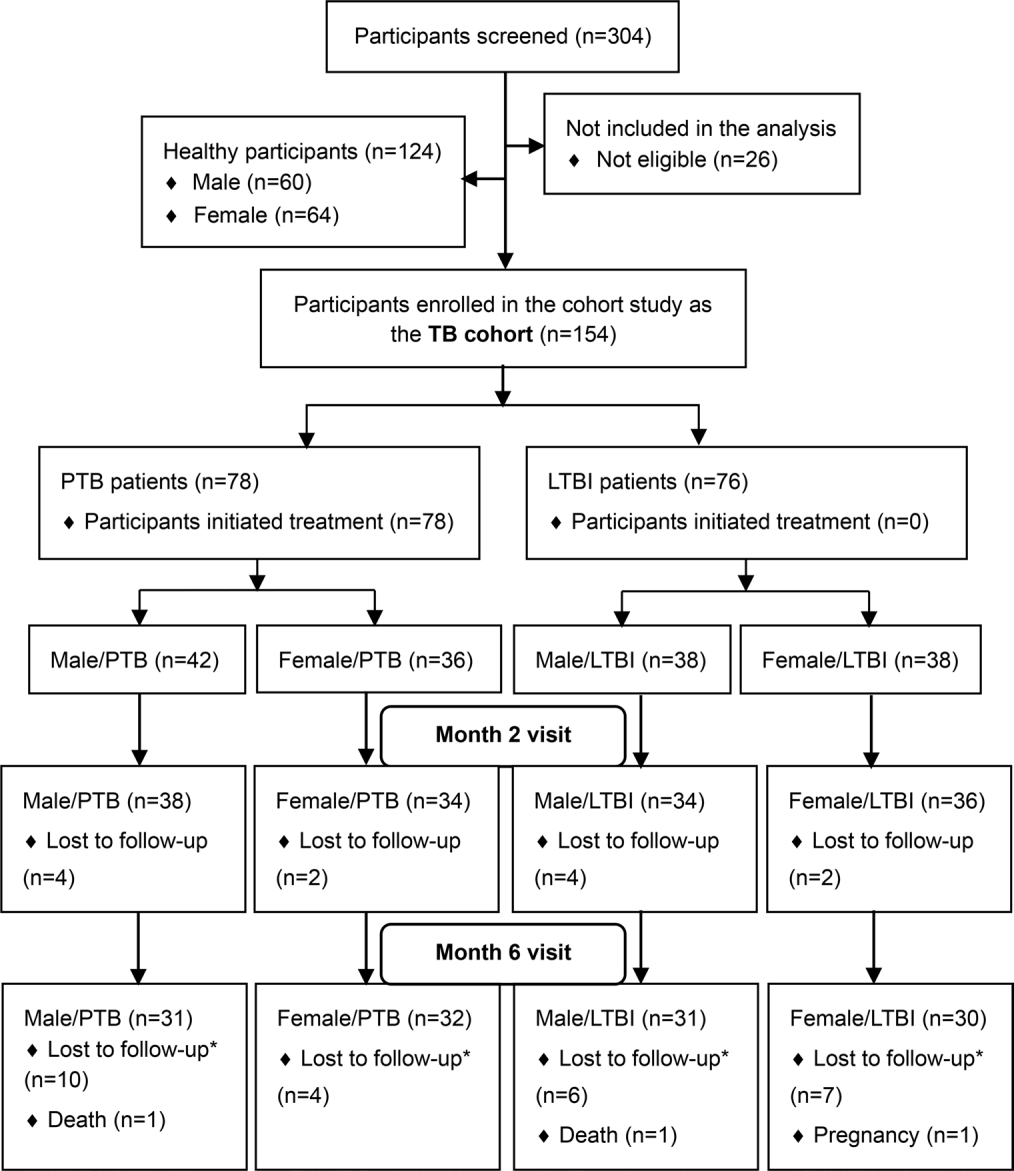
Study flow chart. Asterisks represent that the number in parenthesis indicates that Lost to Follow-up is cumulative (e.g., number of participants who missed their study visits on Month-2 and Month-6).

In total, 304 adults were screened for study eligibility, of whom 154 were enrolled (Figure 1). The remaining individuals (*N* = 150) were not eligible because of HIV/TB coinfection, drug resistance to standard TB regimen, or severe anemia (*N* = 26) or were healthy donors (*N* = 124) who tested negative for latent TB infection (LTBI) and HIV infection at screening. Participants who enrolled for follow-up visits were either index TB patients with PTB (*N* = 78) and naive of treatment, or their contacts with LTBI (*N* = 76), both referred to as the “TB cohort” in Figure 1. The TB cohort was followed for 6 months with 3 visits: baseline (day 0), month 2, and month 6 of treatment. In total, 27 participants (18% of PTB and 17% of LTBI) were lost to follow-up (LTFU). LTFU was defined as participants who missed their study visit at the indicated window. LTFU at the end of treatment was higher in males with PTB (10; 23.8%) compared with females with PTB (4; 11.1%) (Figure 1). Similarly, we observed 2 deaths during the study, both of males, one with PTB and one with LTBI, corresponding to a male mortality rate of 2.5% versus zero for females. None of the participants with LTBI developed PTB during the 6-month follow-up.

### Differences in baseline sociodemographic, behavioral, and clinical characteristics between males and females.

Characteristics of the study participants are summarized in Table 1. Among male participants, young adults between 25 and 34 years old were most represented. Female participants were predominantly younger and aged between 18 and 24 years. Differences in age distribution between males and females were significant in the PTB group but not in the LTBI or the healthy group. Most participants, both males and females, in the TB cohort (PTB and LTBI groups) were married. Illiteracy rates were higher in females compared with males with PTB. The proportion of females with PTB who had a university degree was lower than that of females in the other groups. Females also earned less income than males across all study groups, but this difference was statistically significant only in the PTB group. Notably, nearly 95% of females in the PTB group earned less than the minimum wage in Mali (about US $63/mo). Opposite trends were observed for family size: females had substantially higher median family size compared with males for all study groups. Based on self-report, 45.2% of males with PTB were smokers and 23.8% drank alcohol; only 5.3% of males with LTBI reported drinking alcohol and 39.5% were smokers. The calculated average pack years for males who smoked was 8.6 ± 8.6 for males with PTB, 5.1 ± 4.4 for males with LTBI, and 7.7 ± 13.9 for healthy males. No females reported smoking, and only 1 healthy female reported drinking alcohol, confirming gender differences in smoking and alcohol drinking behaviors in our study population. Only 3 females reported the use of fertility treatment, while 30.5% of females with PTB and 39.5% of females with LTBI used contraception. History of diabetes and history of gastritis were more common in females than in males with PTB. Likewise, abnormal BMI (both low and high) was predominant among females in all study groups.

During the medical exam, Bacille Calmette-Guérin vaccination determined by the presence of a scar on one of the forearms was common, regardless of sex. Among the PTB group, fever, defined as body temperature ≥37.5°C, was noted in 27.8% of females and 19% males. Abnormal radiological findings, including unilateral and bilateral lung infiltrates, were found in both males and females. The same observation was true for the presence of cavities in the lungs. Furthermore, microbiological data in the PTB group revealed that males had many acid-fast bacilli (AFB) in their sputum (88.1%) compared with females (72.2%) at baseline, but this was not statistically significant in a univariate analysis. To rule out the possibility that the observed trend for higher mycobacterial load in males was associated with *M. tuberculosis* genotypes, we performed spoligotyping, which did not reveal evidence of any genotypes of *M. tuberculosis* complex preferentially selected in males compared with females (Supplemental Table 1; supplemental material available online with this article; https://doi.org/10.1172/jci.insight.200877DS1). Our data revealed similarities and divergences between males and females with PTB at baseline in the absence of treatment.

### Testosterone in males, but not estradiol in females, is altered during active PTB.

**Figure 2:**
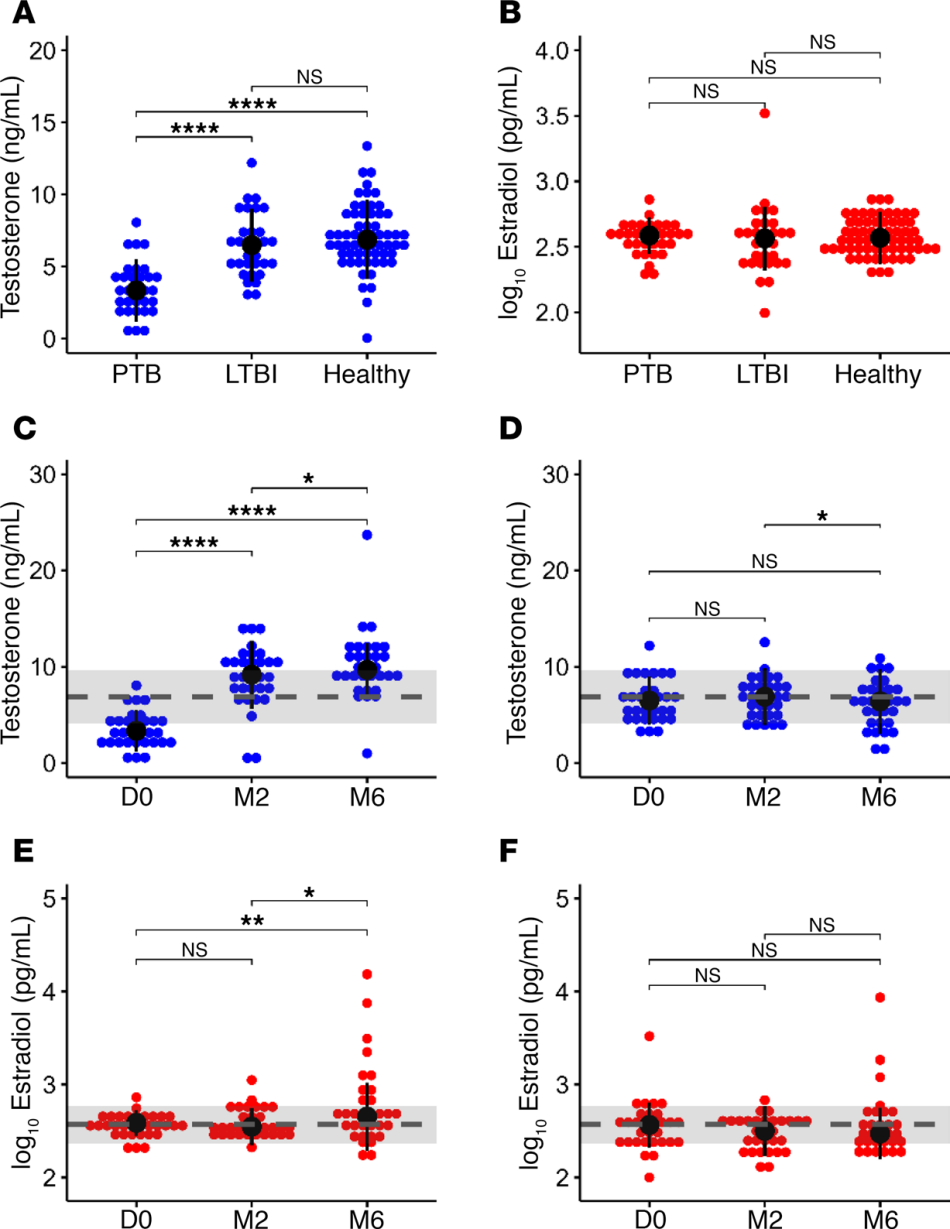
Changes in gonadal steroid concentrations in males and females by TB status and during treatment. (**A**) Testosterone concentrations in males at baseline in individuals with active pulmonary TB (PTB) and latent TB infection (LTBI) and healthy controls (Healthy). (**B**) Log-transformed estradiol concentrations in females with PTB and LTBI and healthy controls before commencing treatment. (**C** and **D**) Longitudinal assessment of testosterone concentrations in males with PTB (**C**) or LTBI (**D**) at different times points over a 6-month period. (**E** and **F**) Concentrations of estradiol on a log scale over time during the study period in females with PTB (**E**) or LTBI (**F**). Individual values for each participant and median ± interquartile range (IQR) are shown. Wilcoxon’s rank-sum test was used for group comparisons for **A** and **B**. Wilcoxon’s matched-pairs signed-rank test was used for comparison between time points for **C**–**F**. Dashed lines and gray zones represent median concentrations and IQR, respectively, of gonadal steroids in healthy donors of similar age and sex. *P < 0.05; **P < 0.001; ****P < 0.00001.

To determine whether TB status affected secretion of gonadal steroids, total testosterone in males and estradiol concentrations in females were measured during the 6-month study period. In males with PTB, testosterone concentrations were suppressed compared with those in either males with LTBI or healthy males (Figure 2A). In contrast, TB status did not impact estradiol concentrations in females (Figure 2B). Longitudinal assessment of gonadal steroid concentrations over the 6 months of standard TB treatment showed that in males with PTB the reduction of testosterone concentrations at baseline was reversed to nearly normal levels by month 2 of treatment, which corresponded to the end of the intensive treatment phase (Figure 2C). In contrast, testosterone concentrations in the LTBI group remained similar to those in healthy males (Figure 2D). Changes in estradiol concentrations in females with PTB were only detectable at month 6 of treatment, corresponding to the end of the maintenance phase of treatment (Figure 2E). This is in sharp contrast to females in the LTBI group, for whom no change was observed over time (Figure 2F). A direct 2-by-2 comparison of gonadal steroid concentrations between PTB and LTBI and by sex can be found in Supplemental Figure 1. The data revealed striking changes in testosterone concentrations in response to PTB treatment in males but also highlighted substantial divergences in testosterone concentrations between patients with PTB compared with those with LTBI.

### Testosterone, but not estradiol, is associated with mycobacterial burden.

**Figure 3:**
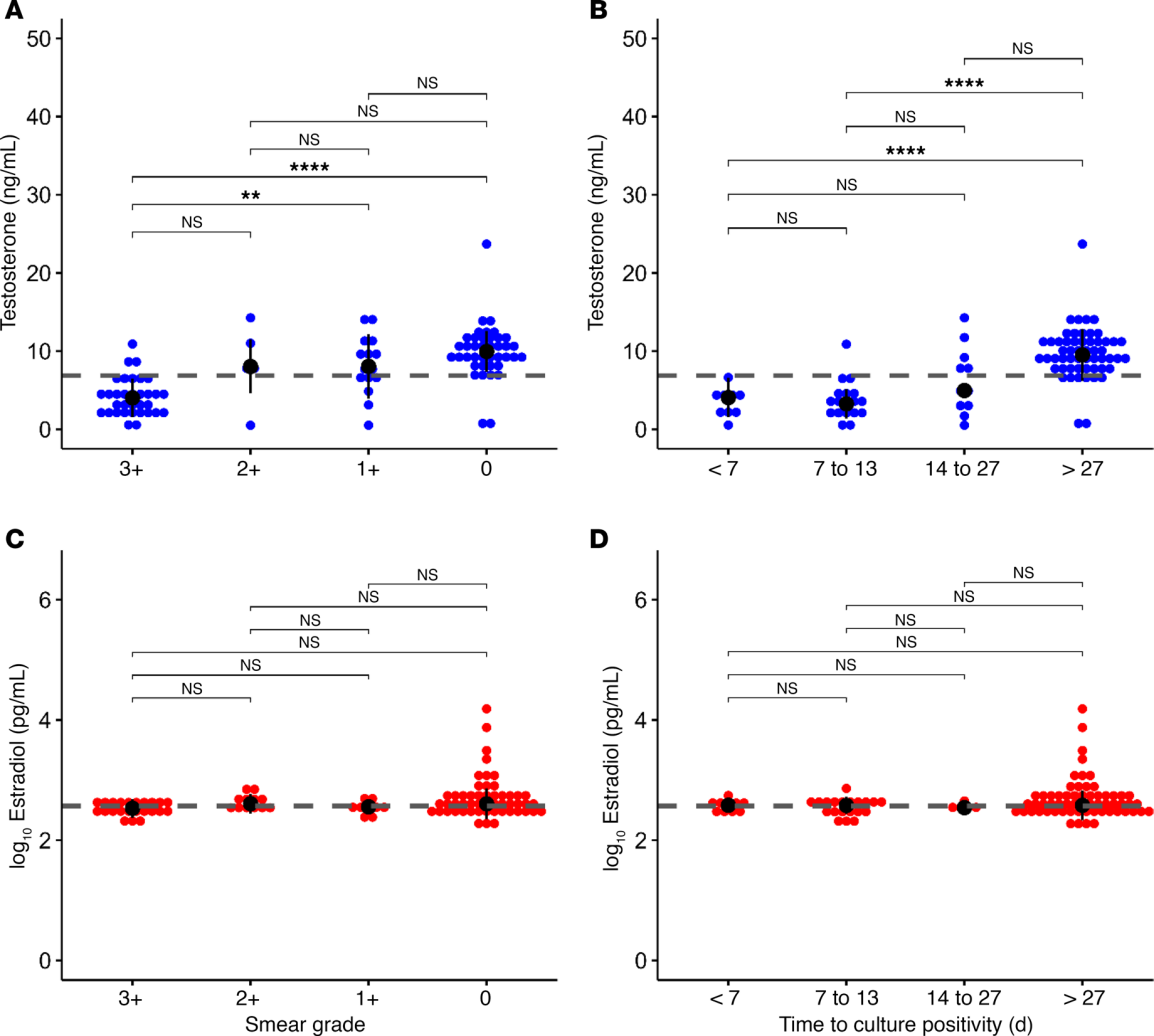
Relationship between gonadal steroids and mycobacterial burden by smear microscopy and culture in both sexes. (**A** and **B**) Smear grade by microscopy (0, no AFB; 1+, few AFB; 2+, moderate AFB; 3+, many AFB) (**A**) and time to culture positivity in days (**B**) on the *x* axes as a function of testosterone concentrations in males on the *y* axis. (**C** and **D**) Assessment of mycobacterial burden (smear grade and time to culture positivity) on the *x* axis in relation to log estradiol concentrations in females on the *y* axis. Smear grade = 3+ and time to culture positivity < 7 represent microbiological data of individuals naive of treatment. Conversely, smear grade = 0 and time to culture positivity > 27 correspond to microbiological results of participants with successful treatment outcome (no AFB). Individual values for each participant and median ± IQR are presented. Wilcoxon’s rank-sum test was used for different comparisons shown in **A**–**D**. Dashed lines show median value obtained from age- and sex-matched healthy donors. **P < 0.001; ****P < 0.00001.

To assess whether testosterone concentrations in males were associated with mycobacterial burden, we compared testosterone concentrations between males with PTB with different smear grades and time to culture positivity, which are both indicative of mycobacterial load in the lungs, irrespective of the treatment status. Males with the lowest concentrations of testosterone had the highest smear grade (i.e., 3+), while males with no detectable AFB who were successfully treated had the highest concentrations of total testosterone (Figure 3A). The lowest time to culture positivity (i.e., <7 days), which is the shortest time it takes for mycobacteria to grow in vitro, was associated with having lower testosterone concentrations in males with PTB (Figure 3B). To ensure that the negative relationship observed between testosterone and mycobacterial burden in participants with PTB was not influenced by age, we stratified our PTB cohort in 2 groups, young (less than 35 years old) and old (35 years or older), as shown in Supplemental Figure 2. We did not detect a difference in testosterone concentrations by age (Supplemental Figure 2A). Similarly, a correlation was not observed between testosterone and age (Supplemental Figure 2B). In contrast to testosterone, no association was observed between concentrations of estradiol and smear grade (Figure 3C) or time to culture positivity (Figure 3D) in females. These data indicate an inverse association between testosterone concentrations in males, but not estradiol concentrations in females, and mycobacteria load in the lungs.

### Testosterone is a predictor of treatment outcomes in males.

**Figure 4:**
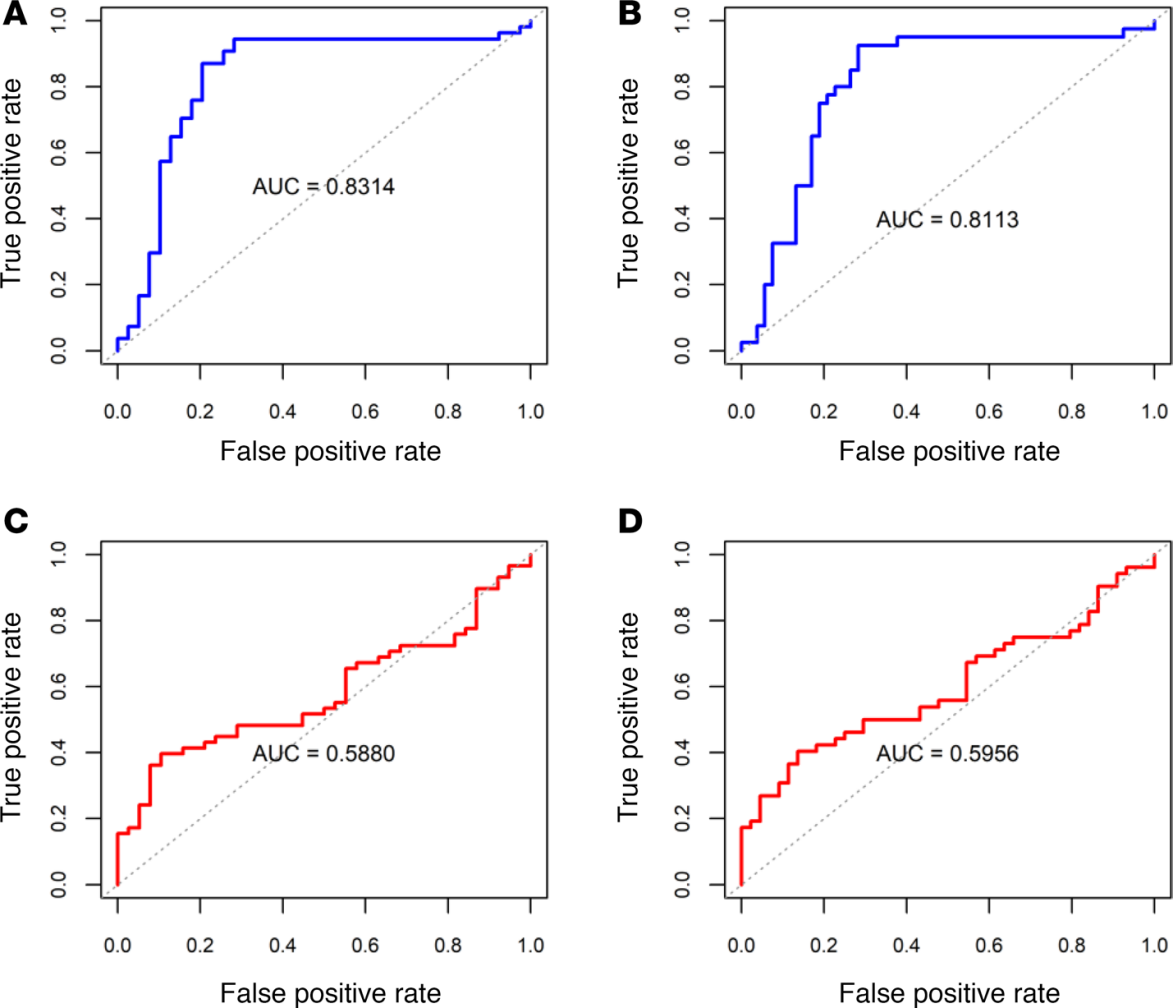
Receiver operating characteristic curves depicting the relationships between concentrations of gonadal steroids and mycobacterial clearance in males and females. The *x* axes show sensitivity, also known as false-positive rate, of testosterone (blue) or estradiol (red) concentrations for predicting smear grade (**A** and **C**) or time to culture positivity (**B** and **D**). The *y* axes represent (1 – specificity), which corresponds to the true-positive rate for the same prediction. The area under the curve (AUC) value for each prediction is shown, with a perfect relationship having an AUC equal to 1. Dashed lines represent the diagonal line of the curve. The closer a graph to the diagonal, the less accurate a prediction will be.

To determine the effect of the negative association between concentrations of testosterone and mycobacterial burden on treatment outcomes, as defined by culture or smear negativity at month 2 or month 6 of treatment, we used generalized linear mixed regression models, adjusting for potential covariates in a sex-specific manner (i.e., age, BMI, smoking [males], alcohol drinking [males], and contraceptive use [females]). A 1-unit increase in testosterone concentrations increased the odds of culture negativity by 58% (*P* < 0.0001) and of smear negativity by 50% (*P* < 0.0002) in males, suggesting a link between testosterone and treatment outcomes (Table 2). In contrast, a log unit increase in estradiol concentrations was not associated with the odds of treatment outcomes in females (Table 2). To determine whether testosterone was predictive of treatment outcomes in males with PTB, receiver operating characteristic curves were computed and revealed area under the curve (AUC) values of 0.8314 and 0.8113 for the relationship between testosterone and smear grade and culture negativity, respectively, for males (Figure 4, A and B). Conversely, estradiol concentrations were not predictive of treatment outcomes, as indicated by AUC values of 0.5880 and 0.5956 for smear grade and culture negativity, respectively (Figure 4, C and D). These data suggest that serum concentration of testosterone is a biomarker for successful PTB treatment outcome in males.

### Testosterone is negatively correlated with circulating inflammatory cytokine levels in males.

**Figure 5:**
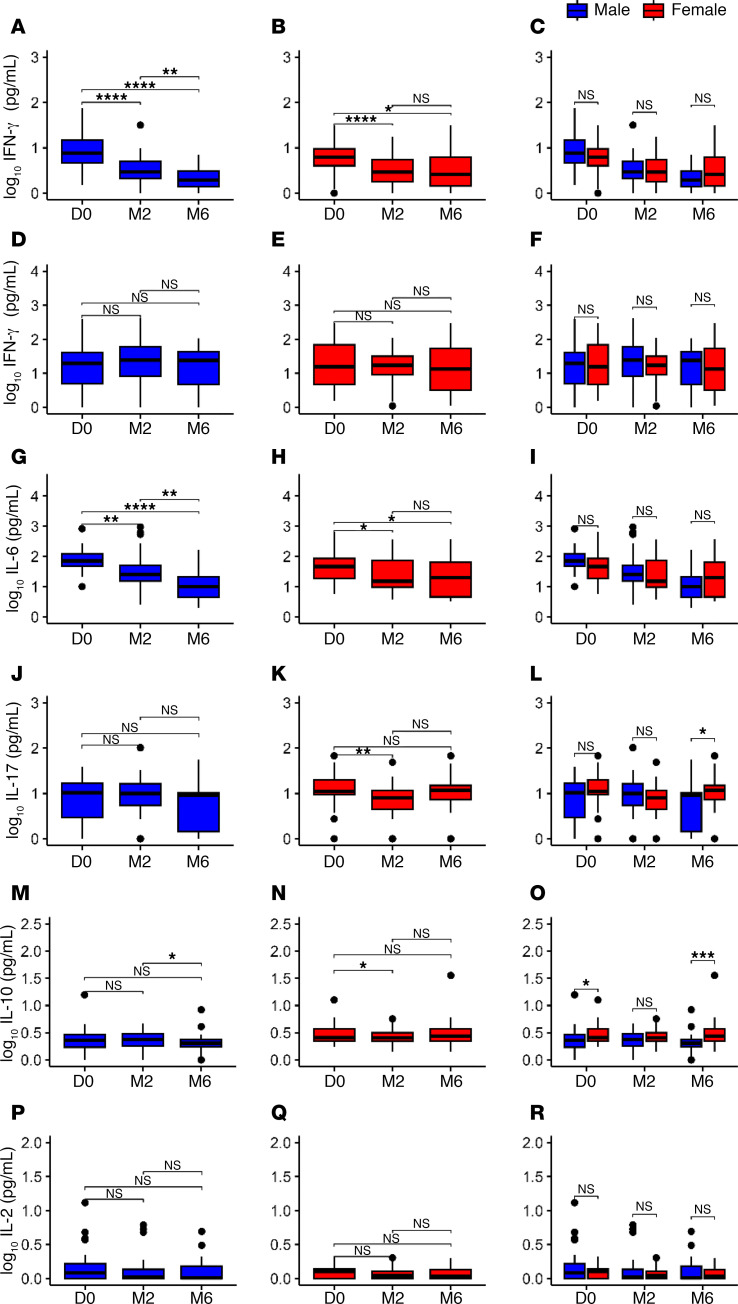
Longitudinal changes in inflammatory cytokine concentrations by sex during treatment for TB. The *x* axes show study visits (baseline, month 2, and month 6), and the *y* axes represent log-transformed cytokine concentrations in the serum. The first 2 columns represent longitudinal assessment of the changes in cytokine concentrations in males (blue box plots) and in females (red box plots) during the study (baseline, month 2, and month 6). The last column shows the comparison of cytokine concentrations between males and females. For each box-and-whisker plot, bounds of the boxes are quartile 1 (25th percentile) and quartile 3 (75th percentile), the line within the box represents the median value, whiskers show maximum and minimum values, and the dots outside the box are outlying values. Wilcoxon’s matched-pairs signed-rank test was used for between-time-point comparisons shown in **A**, **B**, **D**, **E**, **G**, **H**, **J**, **K**, **M**, **N**, **P**, and **Q**. Wilcoxon’s rank-sum test was used for sex comparisons within each time point for **C**, **F**, **I**, **L**, **O**, and **R**. *P < 0.05; **P < 0.001; ***P < 0.0001, ****P < 0.00001.

To consider the mechanisms by which greater testosterone is associated with favorable treatment outcome, we assessed the relationship between gonadal steroids and systemic inflammatory cytokines, including IFN-γ, TNF, IL-6, IL-2, IL-10, and IL-17. Dynamic changes in cytokine levels during PTB treatment were analyzed by comparison of levels found in males and females. There was a longitudinal decline in IFN-γ and IL-6 levels in both males and females (Figure 5, A, B, G, and H). Conversely, TNF and IL-2 levels did not change during follow-up in either sex (Figure 5, D, E, P, and Q). Nonetheless, sex differences in the levels of IFN-α, TNF, IL-6, and IL-2 were not observed at any of the time points investigated during treatment (Figure 5, C, F, I, and R). In contrast, IL-17 and IL-10 displayed notable sex-differential patterns (Figure 5, J–O), in which IL-17 levels were lower at month 2 of treatment compared with baseline in PTB females, but not in males (Figure 5K). Higher levels of IL-17 at month 6 of treatment were observed in females compared with males (Figure 5L). IL-10 levels significantly decreased in males from month 2 to month 6, which corresponded to the maintenance phase of treatment (Figure 5, M and O). In contrast, changes in IL-10 levels in females were only observed during the intensive treatment phase between baseline and month 2 (Figure 5N). This dynamic change in IL-10 levels by sex led to higher IL-10 levels in females compared with males at baseline and at month 6 (Figure 5O). Such sex-specific variation in IL-10 levels was also evident when we performed a temporal analysis across the 3 time points (Supplemental Figure 3).

**Figure 6:**
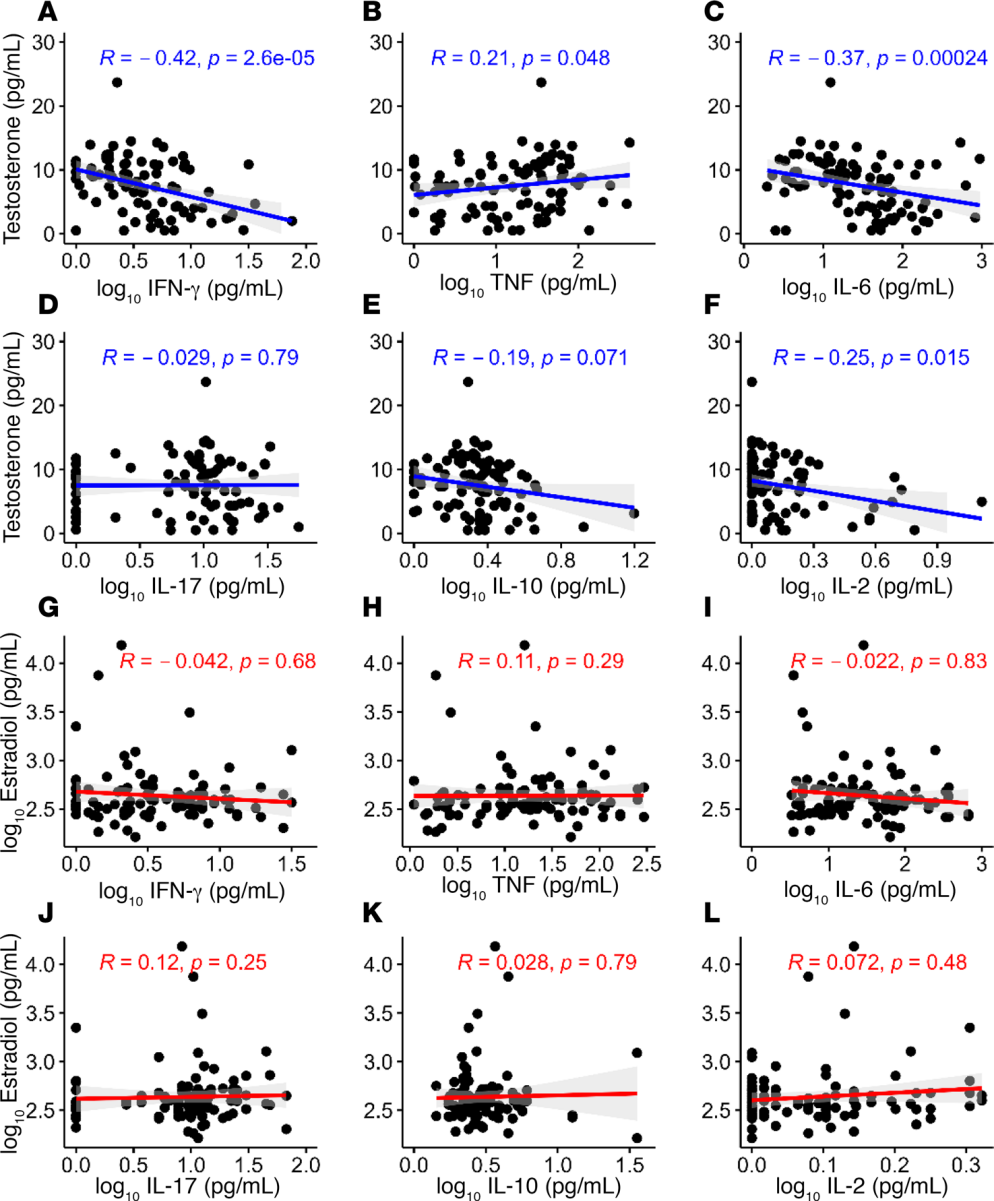
Relationship between gonadal steroid and inflammatory cytokine concentrations during TB. (**A**–**L**) Correlation analysis (Spearman’s test) between log-transformed cytokine concentrations on the *x* axis and testosterone and estradiol (in log) on the *y* axis. Each dot represents a study participant. Linear correlation lines are indicated in blue for testosterone in males and in red for estradiol in females. Similarly, the 95% confidence interval of the correlation line is shown in gray. Numbers at the top of each graph represent the coefficient of correlation (*r*) and *P* value of the relationship. Spearman’s rank method was performed to correlate gonadal steroid and inflammatory cytokine concentrations for all panels of the figure.

Concentrations of gonadal steroids were correlated with specific inflammatory markers. Testosterone concentrations in males were negatively correlated with IFN-γ, IL-6, and IL-2, but not IL-10 and IL-17 (Figure 6, A–F). The correlation between testosterone and TNF was borderline significant but positive (Figure 6B). Concentrations of estradiol in females did not correlate with any of the cytokines tested (Figure 6, G–L). These data suggest a possible crosstalk between inflammatory responses and androgens with testosterone playing a potentially antiinflammatory role during PTB.

## Discussion

We conducted a longitudinal, prospective, cohort study in Bamako, Mali, to understand sex differences in the association of gonadal steroid concentrations with TB disease and treatment outcomes. The association between gonadal steroids in male and female participants and TB disease states revealed that PTB was associated with significantly lower concentrations of testosterone in males but not estradiol in females. Low testosterone concentrations were associated with high smear grade and shortest time to culture positivity among males. A 1-unit increase in testosterone level was predictive of cure in males receiving TB treatment. The same relationship was not apparent between estradiol and TB outcomes in females. We further observed sex differences in circulating IL-17 and IL-10 levels, with changes in INF-γ and IL-6 levels conserved in both sexes. Our findings indicate a potential role for testosterone in TB pathogenesis and, more importantly, its potential utility as a biomarker to monitor treatment outcomes.

Gonadal steroids are known to act on various cells of the innate and adaptive system (19). The effects of testosterone on immune cells can be cell specific, but often facilitate antiinflammatory responses, ranging from blocking immune cell activation or proliferative capacity to modulating expression of cytokines or lineage-defining transcription factors (19, 20). Reduced concentrations of testosterone in males with TB who were naive of treatment have been reported previously in sex-disaggregated TB cohorts (21, 22), as well as in mixed cohorts (23, 24). Two hypotheses have been postulated to explain lower testosterone levels in males with PTB. First, reduced testosterone concentrations could be a consequence of a general alteration of men’s health during TB disease, impacting the metabolism of androgens. For example, reduction of the levels of dehydroepiandrosterone (DHEA), a precursor of testosterone with known immunomodulatory properties, has been reported during TB (22, 25). Similarly, hypogonadism characterized by low testosterone levels has been reported in other infectious diseases in males, such as severe COVID-19 and HIV-1 infection (26, 27). Secondly, alteration of testosterone levels in males with PTB could be mediated by the effects of endogenous inflammatory cytokines, such as IFN-γ, TNF, and IL-6, on the reproductive system. Accordingly, in vitro treatment of Leydig cells, which are the testosterone-producing cells of the testes, with IFN-γ or TNF reduces testosterone synthesis (21). Our observation of an inverse correlation between testosterone concentrations and inflammatory cytokines, such as IFN-γ, IL-6, and IL-2, which can be classified as Th1 cytokines, is consistent with the hypothesis that activation of inflammatory immune responses can inhibit testosterone secretion. In our study, low testosterone concentrations were associated with heightened inflammatory responses and greater TB disease in males, suggesting a potential role for androgens in the pathogenesis of TB.

During the longitudinal analysis, low levels of testosterone were nearly restored to physiological levels by the end of the intensive phase of treatment (month 2). Such an increase in testosterone levels was inversely associated with mycobacterial burden and was a strong predictor of cure after treatment, which was not observed with estradiol in females. Sex-specific, longitudinal assessment of gonadal steroids during TB treatment is scarce and is often limited to 2 time points (before and after treatment) or limited by comparison of treatment success and failure, using a pooled dataset of both sexes (28). Nonetheless, data on variation of DHEA levels during TB treatment are comparable to our findings. Specifically, work done by Tsegaye and colleagues showed an increase in both testosterone and DHEA at the end of the treatment (23), but also reported normalization of cortisol and DHEA levels as well as their ratio in patients who had achieved full clinical recovery from PTB (29, 30). Our data are also consistent with a recent report based on a 10-year follow-up of a non-TB cohort that observed that gonadal steroid concentrations may be predictors of health and disease status in males but not in females (31). We postulate that testosterone concentrations could potentially be used to monitor TB treatment outcomes among males.

Like testosterone, estradiol has been shown to have immunoregulatory activities in various cells expressing estrogen receptors (19). However, the effects of estradiol can be paradoxical (proinflammatory or antiinflammatory) depending on the dose, target cells, and timing (32). We did not observe an association between estradiol and TB status in females, which is in sharp contrast to testosterone in males. We found a modest increase in estradiol levels from baseline to the end of treatment (month 6) in PTB but not in LTBI. However, changes in estradiol levels were not associated with smear grade. In contrast to testosterone, variations in estradiol levels did not predict cure and were not correlated with inflammatory cytokines levels. Our data suggest that estradiol may be involved in TB pathogenesis but not in the host response to treatment. This hypothesis is supported by experimental findings derived from a mouse model of infection with *Mycobacterium avium*, a non-tuberculous mycobacterium, which compared the phenotypes of ovariectomized female mice versus sham-operated mice (33). In this study, ovariectomized mice had higher mycobacterial loads in the lungs compared with control mice. In addition, treatment of ovariectomized mice with estradiol reduced mycobacterial load in the lungs at the same levels as in sham-operated mice, indicating a potential role of estradiol in enhancing female resistance to TB. These findings are corroborated in human studies showing that post-menopausal women, who presumably have low levels of estradiol, have an increased risk of developing TB caused by *M. avium* (34). Likewise, low serum levels of estradiol were found to be an independent predictor (AUC = 0.947) of *M. avium* complex–induced lung disease, which was not the case with PTB caused by species of *M. tuberculosis* complex in our investigation (35). Like our work, Tsegaye and colleagues did not find a difference in estradiol levels between PTB, LTBI, and healthy participants. They did, however, find an increase in estradiol levels at the end of treatment, as in our study. Thus, these inconsistencies between studies suggest that future investigations are needed to understand the role played by estradiol during TB caused by tuberculous versus non-tuberculous mycobacteria.

Among the 6 cytokines studied, we found sex-specific differences for 2 cytokines, IL-17 and IL-10. The first is a prototypical Th17 cytokine, while the second is a major immunoregulatory cytokine with patent antiinflammatory activities. We found higher levels of IL-17 in females than in males at month 6, while males and females exhibited differential IL-10 levels at both baseline and month 6. The importance of these two cytokines with opposing functions (proinflammatory versus antiinflammatory) in TB has been extensively reviewed (36, 37). Hertz and colleagues found higher expression of IL-17A in the lungs of female mice compared with males early after infection with the Beijing H8N8 strain, which is considered as a hypervirulent mycobacterial strain in rodents. Such induction was found to be consistent with heightened induction of IL-17–promoting cytokines, such as IL-23 and IL-1β, in females relative to males throughout the course of disease, irrespective of the infective mycobacterial strains used, indicating a potential contribution of the Th17 axis in sex differences in the context of TB (18). In contrast to IL-17, the role of IL-10 in TB is less well understood. A recent study in cynomolgus macaques suggested a potential immunoregulatory role of IL-10 in TB, as neutralization of IL-10 early during infection led to notable immunological changes such as lower lung inflammation and increased cytokine production within the granuloma and the lymph nodes of those animals compared with controls (38). Similarly, ablation of IL-10 in B cells promotes resistance to *M. tuberculosis* in mice with a stronger effect in males compared with females (39). Because IL-17 and IL-10 have both been implicated in sex differences in other bacterial infections (40, 41), it is likely that the two cytokines mediate sex differences in TB outcomes. Additionally, key cytokines important to Th1 immunity to TB, such as IFN-γ, IL-6, and IL-2, were negatively correlated with testosterone. These data are consistent with the finding that testosterone replacement therapy in men with testosterone deficiency and type 2 diabetes decreases the production of the proinflammatory cytokines IL-1β, IL-6, and TNF by APCs (42), but also increases expression of TNF and IL-1β upon pharmacological castration. The inhibitory effects of testosterone on cytokine mRNA expression can be direct by repression of transcription via androgen response elements in the promoters of cytokine genes, such as for the murine *Ifng* gene (43). Effects of testosterone on cytokines can also be indirect, such as through induction of IL-10 expression in CD4^+^ T cells (44) or by upregulation of the phosphatase Ptpn1, which blocks cytokine signaling necessary for Th1 differentiation (45). Together, our data indicate potential bidirectional communication between testosterone and proinflammatory cytokines in the context of PTB to facilitate optimal Th1 immunity and maintain homeostasis after treatment.

Our study has many strengths, including (a) use of age- and sex-matched control groups composed of healthy individuals and LTBI individuals; (b) longitudinal measurement of gonadal steroids and inflammatory cytokines before treatment (baseline), during treatment (month 2), and at the end of treatment (month 6); and (c) meticulous collection of non-biological variables allowing for multiparameter regression analyses to control for clinical and behavioral covariates. Some limitations include that we did not measure testosterone in females and estradiol in males because of their intrinsic low concentrations, which fall below the detection limits of the assays used. Use of a more sensitive assay such as mass spectrometry would have allowed us to compute testosterone and estradiol ratios for each sex. Second, we did not know female participants’ phase of the menstrual cycle when estradiol was measured. Thus, we cannot rule out that our data may be affected by intrinsic differences in estradiol concentrations between the luteal and follicular phases of the menstrual cycle. Third, we focused on the 2 dominant gonadal steroids, testosterone and estradiol, but analysis of additional hormones such as DHEA, cortisol, progesterone, and thyroid hormones would be a more comprehensive assessment of the changes in hormonal milieu in both sexes in the context of TB. Fourth, our investigation was limited to an exploratory cohort; more investigation using a validation cohort will be needed to confirm temporal variations observed in both testosterone and cytokine concentrations. Lastly, we focused on patients infected with drug-susceptible mycobacteria; thus, at the end of the study, 100% were cured (i.e., smear or culture negative). It will be necessary to ascertain whether the relationship between testosterone and treatment outcomes found in this study is consistent in a cohort of drug-resistant or non-compliant PTB patients with treatment failure. Despite these limitations, our data suggest that testosterone in males, but not estradiol in females, is a strong predictor of cure during standard PTB treatment as well as a correlate of antiinflammatory responses. Future studies are warranted to understand the role of testosterone in immunity and response to treatment during TB.

## Methods

### Sex as a biological variable

This study was specifically designed to consider sex as a biological variable in the pathogenesis and treatment outcomes of TB. Thus, adult males and females of similar ages were enrolled in the study. All demographic, clinical, and laboratory data are presented for both sexes with the goal of yielding data that are expected not only to be relevant to both males and females but also to reveal sex-specific differences.

### Participants and clinical procedures

We conducted a longitudinal, prospective cohort study from October 2020 to July 2022 by screening and enrolling study participants at the 6 referral TB diagnosis and treatment centers and the Department of Pneumophtisiology of the Teaching Hospital of Point-G in Bamako, the capital city of Mali. Participants were selected based on sputum microscopy results, for index PTB participants; or as family members or friends of the same sex and similar age (±3 years) who were in contact with an index PTB participant, as defined by WHO criteria, for the control. PTB participants who were naive of treatment, between 18 and 55 years old, were invited to participate in the study. Eligible subjects received detailed information on study objectives, duration, number of visits, blood and sputum volume, chest x-rays, risks, and benefits. Subjects who consented to participate were screened for inclusion criteria including hemoglobin level >8 g/dL, absence of pregnancy for females, negative HIV serology, and sputum culture positive for a species of *M. tuberculosis* complex that is sensitive to all first-line anti-TB drugs for index cases, or a positive QuantiFERON test (QIAGEN) along with absence of radiological findings for individuals with LTBI. Participants who tested negative for QuantiFERON and HIV were considered “healthy” controls and were not followed as part of the longitudinal cohort. Each participant underwent a structured questionnaire about his or her medical history, use of contraception in females, and assessment of current symptoms. Clinical data, such as temperature, weight, and height, were collected. A physical exam was also performed. Blood was collected as well as 5 mL sputum samples for 2 consecutive days at baseline for laboratory tests. Chest x-ray films were analyzed to assess presence or absence of lesions. PTB and LTBI participants were followed for 6 months, during which clinical and biological parameters were assessed. All participants with PTB received free anti-TB drugs at their diagnostic center, according to the national TB program guidelines (2RHZE/4RH), as follows: 2 months of rifampicin (R) + isoniazid (H) + pyrazinamide (Z) + ethambutol (E) (intensive phase) followed by 4 months of rifampicin (R) + isoniazid (H) (maintenance phase). All clinical data were entered into the Research Electronic Data Capture (REDCap) system using electronic case report forms.

### Laboratory procedures

#### HIV diagnosis.

HIV infection was diagnosed using the rapid serological Alere Combo kit (Abbott) for HIV-1/HIV-2 antigen and antibody detection. Any individual with a positive result was referred to an HIV Diagnosis and Treatment Center for confirmation of the serology and treatment, according to the national guidelines.

#### Microbiology for diagnosis of active PTB.

Sputum smear microscopy using auramine-rhodamine staining, Xpert MTB/RIF (*M. tuberculosis*/rifampicin; Cepheid) testing, and culture with liquid and solid media were performed on all samples at baseline. Participants were asked to bring a second sample on day 1 to confirm the diagnosis. Indirect microscopy was performed to determine the presence of mycobacteria, as previously described (46). Smears were prepared using concentrated pellets of processed sputum samples. Then the smears were air-dried, heat-fixed, stained with Auramine O, and examined using a fluorescent microscope. Smear grade was established based on the International Union Against Tuberculosis and Lung Diseases grading criteria, which are: negative (no AFB seen, or 0), scanty AFB (1–9 AFB per 100 immersion fields), few AFB (10–99 AFB seen in 100 fields, or 1+), moderate AFB (1–10 AFB seen per field, or 2+), and many AFB (>10 AFB seen per field, or 3+) (47). To ensure accuracy of the grading, smears were read independently by 2 operators; in cases of discrepancy, a third operator was introduced as the tie breaker. Xpert MTB/RIF testing was performed for molecular detection of mycobacterial genome, as previously reported by our research group (48). This method allows simultaneous detection of both genomic DNA of *M. tuberculosis* complex (MTBC) and the resistance to rifampicin based on the presence or absence of the wild-type *rpoB* gene.

#### Culture and drug susceptibility testing.

Culture and drug susceptibility testing (DST) were conducted as follows: Sputum samples were digested and decontaminated using the standard *N*-acetyl-l-cysteine/4% NaOH solution, concentrated by high-speed centrifugation (3,396*g*), and inoculated on both liquid and solid media, namely the Mycobacterium Growth Incubator Tube (BBL MGIT, Becton Dickinson) and the Middlebrook 7H11 Agar and Selective 7H11 Agar media. At the same time, an aliquot of concentrated sputum was prepared for auramine-rhodamine staining (BBL, Becton Dickinson) to determine baseline smear grade. Continuous monitoring of cultures was performed for a total of 6 weeks or 42 days at 37°C using the BACTEC MGIT 960 machine until presence or absence of mycobacterial colonies. Speciation of recovered MTBC isolates was based on a combination of fluorescent acid-fast microscopy, colonial morphology, and the use of nucleic acid probes (AccuProbe GenProbe). The recovered isolates from either liquid or solid media were confirmed as MTBC using the SD BIOLINE TB Ag MPT 64 assay or the Capilia TB-Neo assay (Tauns Laboratories Inc.), as previously described (46, 49). Indirect DST was performed on MTBC isolates with an MGIT AST/SIRE System (Becton Dickinson) using streptomycin (S), isoniazid (I), rifampicin (R), and ethambutol (E), and interpretation of the results was based on the US Centers for Disease Control and Prevention recommendations for critical drug concentrations (50).

#### Spoligotyping technique for genotyping MTBC strains.

Spoligotyping is a PCR-based method for simultaneous detection and genotyping of strains of all members of the MTBC. It was performed using a commercially available kit (Isogen Life Science), as previously reported (51–53). Strain comparisons were made with the SPOTCLUST (SpolDB3-based) database following development of the film. Corresponding shared spoligotypes were further defined using the SITVIT (Institut Pasteur de la Guadeloupe) database.

#### Diagnosis of LTBI.

We used the QuantiFERON-TB Gold Plus assay from QIAGEN (QFT Plus, 622120, 622822) for the diagnosis of LTBI. QFT Plus is an IFN-γ release assay, which is based on the detection of IFN-γ (IU/mL) in the supernatant of whole blood stimulated with mycobacterial antigens. The assay was performed on heparinized blood according to the manufacturer’s instructions. Cell supernatants were collected overnight and frozen for batch testing. The enzyme-linked immunosorbent assay (ELISA) of the supernatants was performed with reagents and supplies included in the kits. ELISA plate was read using the SpectraMax Plus 384 microplate reader available in our laboratory. Data were acquired using the SoftMax Pro software, then uploaded into QFT Plus Analysis Software for specific analysis. LTBI cases were identified based on criteria set by the manufacturer.

#### Operational definitions.

In this study, smear positivity was defined as the presence of AFB on sputum smear after auramine-rhodamine staining, which were quantified as few AFB (1+), moderate AFB (2+), and many AFB (3+). Conversely, smear negativity was defined as the absence of AFB (represented as 0) on sputum smear after Rhodamine-Auramine staining. Time to culture positivity was defined as the time it takes for a clinical mycobacterial isolate to grow in a liquid medium (Mycobacterium Growth Incubator Tube [MGIT], Becton Dickinson) seeded inside a BD BACTEC 960 (Becton Dickinson) instrument. Successful treatment outcome in this study regroups patients with bacteriologically confirmed PTB at enrollment who tested negative for MTBC (according to sputum smear or culture) at the end of a 6-month TB regimen.

#### Measurement of gonadal steroids and cytokines.

Serum samples were obtained from blood collected in a BD serum separator tube (367986, Becton Dickinson) and stored in a –80°C freezer until analysis. Samples were gently thawed overnight at 4°C before analysis. We used the testosterone ELISA kit from MyBioSource (MBS494249) for quantitative measurement of testosterone (ng/mL) and the Estradiol ELISA kit from Calbiotech (ES380S) for quantification of estradiol (pg/mL) according to the manufacturers’ instructions using instrumentation and software described above. All samples, calibrators, and controls were run in duplicate. Samples were diluted 1:10 in assay diluent for estradiol measurement. Similarly, serum cytokines were measured using the BD Cytometric Bead Array Th1/Th2/Th17 kits (560484), allowing simultaneous detection of 6 cytokines, namely IFN-γ, TNF, IL-6, IL-17, IL-10, and IL-2, based on procedures described elsewhere (54). Samples were acquired on the BD LSR-II instrument in our laboratory and analyzed using the BD FCAP Array software.

### Statistics

All data were analyzed using R software, version 4.2.2. Testosterone concentrations and log-transformed estradiol levels were compared in a 2-by-2 manner between the healthy, LTBI, and PTB groups, between the different smear grades, and between the different categories of time to culture positivity, using a Wilcoxon’s signed-rank test. Such comparison was done in a sex-specific manner by separation of male and female data. The same approach was to compare testosterone, estradiol, and cytokine concentrations between LTBI and PTB groups and between males and females at each time point of measurement (baseline, month 2, and month 6). Then, the 2-by-2 comparisons of testosterone, estradiol, and cytokine concentrations were performed between the different time points using Wilcoxon’s matched-pairs signed-rank test. Testosterone and estradiol concentrations were correlated with cytokine levels using Spearman’s rank method. Binomial generalized linear mixed regressions were built to determine the effect of changes in testosterone and estradiol concentrations on cure (defined by smear or culture negativity at month 2 or month 6) among PTB cases. The model used to evaluate the effect of testosterone was adjusted for age, BMI, smoking, and alcohol use in men. Similarly, age, BMI, and contraceptive use were considered as covariates and adjusted for women, since only one woman reported drinking alcohol. To evaluate the performance of testosterone and estradiol concentrations in predicting TB cure, the area under the curve values were computed using receiver operator curves. A 2-tailed *P* value less than 0.05 was considered statistically significant.

### Study approval

The clinical protocol of this study was approved by the ethical committee (Comité Institutionnel d’Ethique de la Recherche en Santé et en Sciences de la Vie de l’Université des Sciences, des Techniques, et des Technologies de Bamako) of the University of Sciences, Techniques, and Technologies of Bamako (2019/160/CE/FMOS/FAPH). Written informed consent was obtained from all participants before they underwent study-specific procedures. All clinical and laboratory procedures were conducted according to good clinical practice (GCP) and good laboratory practice (GLP) instructions, respectively.

### Data availability

Values for all data points in figures can be found in the Supporting Data Values file.

## Author contributions

DD, RLM, JLH, CJA, WRB, and SLK designed the study, analyzed and interpreted the results, and wrote the manuscript. BB and AS enrolled and followed the participants for the study, developed the clinical database, interpreted the results, and reviewed the manuscript. IS, MDC, and AD provided a new analytical approach for data analysis, interpreted the results, and reviewed the manuscript. MW, MK, IK, MN, NC, BS, MDC, MP, MS, and BD processed the samples, collected the data, and reviewed the manuscript. SS, S Diallo, MD, YT, S Doumbia, and SLK critically edited and reviewed of the manuscript.

## Conflict of interest

The authors have declared that no conflict of interest exists.

## Funding support

This work is the result of NIH funding, in whole or in part, and is subject to the NIH Public Access Policy. Through acceptance of this federal funding, the NIH has been given a right to make the work publicly available in PubMed Central.

- Fogarty International Center and the Office of the Director of the National Institutes of Health (NIH) through the Office of Research on Women’s Health, under a Career Development Award (K43TW011426) to DD
- Institute for Global Health at the Feinberg School of Medicine of Northwestern University, under the Catalyzer Award to RLM and DD
- 2021 TWAS (The World Academy of Sciences)
- Abdool Karim Award via UNESCO to DD
- National Institute of Allergy and Infectious Diseases grant R37AI167750 to WRB and SLK
- Fogarty International Center research and training grant 5D43TW010350 to RLM

## Supplementary Material

Supplemental data

ICMJE disclosure forms

Supporting data values

## Figures and Tables

**Table 1:**
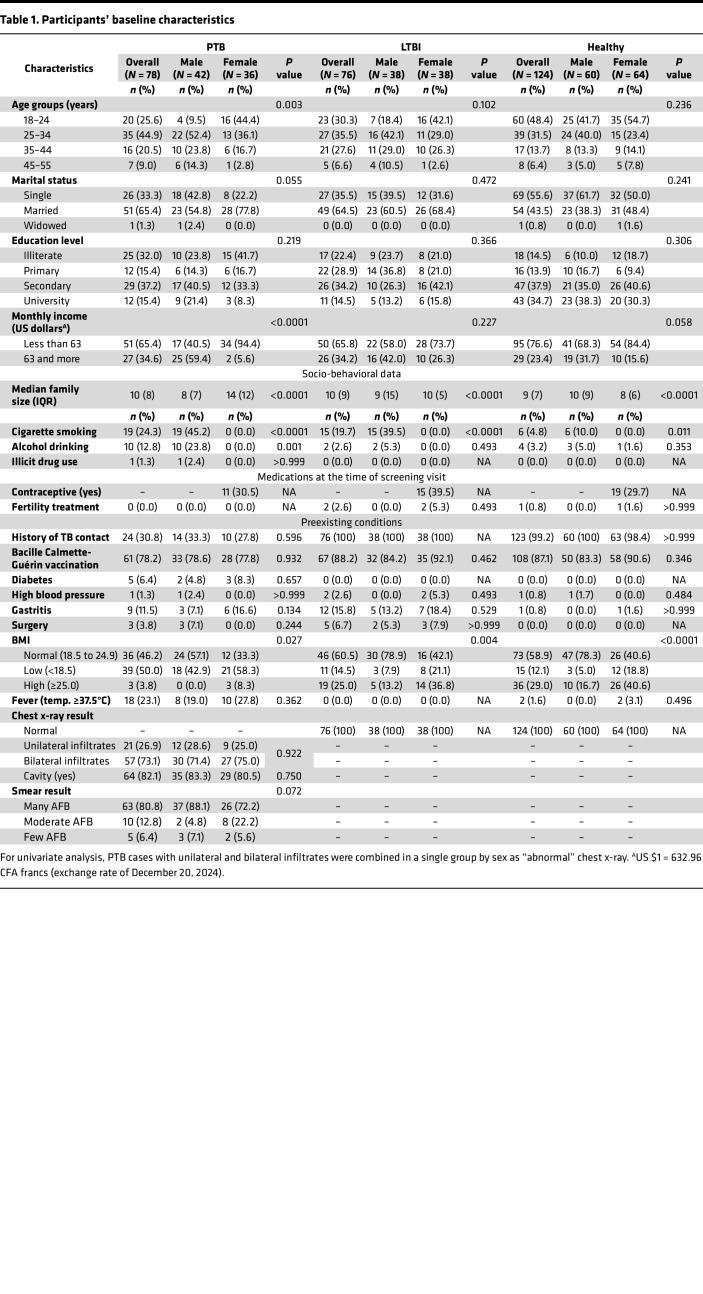
Participants’ baseline characteristics

**Table 2:**
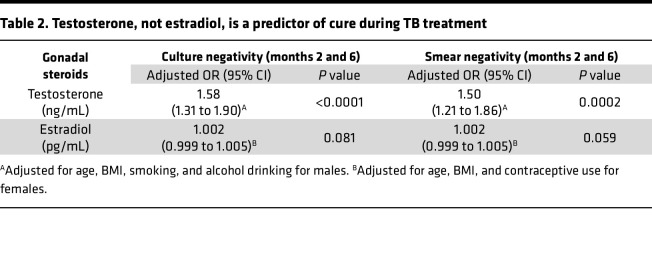
Testosterone, not estradiol, is a predictor of cure during TB treatment

## Version 1 06/16/2026

In-Press Preview

## Version 2 08/10/2026

Electronic publication
