# Supplementary material for: Gonadal steroids as predictors of sex differences in tuberculosis outcomes
Source: JCI Insight. 2026 Jun 16;11(15):e200877. doi: 10.1172/jci.insight.200877 (PMC13464010; doi:10.1172/jci.insight.200877)
Supplement: Supplementary file 1 [file jciinsight-11-200877-s303.pdf]

Supplement 1: Genotypes of mycobacteria isolated among study participants by sex

Lineage (L)	Family	Overall (N=78)	Male (N=42)	Female (N=36)
		# (%)	# (%)	# (%)
Indo-oceanic (L1)	EAI	-	-	-
East Asian (L2)	Beijing	-	-	-
Central Asia (L3)	CAS	-	-	-
Euro-American(L4)	LAM	40 (51.3)	23 (54.8)	17 (47.2)
	Haarlem	3 (3.8)	2 (4.8)	1 (2.8)
	T-Clade	14 (17.9)	6 (14.3)	8 (22.2)
	H37Rv	2 (2.6)	1 (2.4)	1 (2.8)
	S-Clade	5 (6.4)	3 (7.1)	2 (5.6)
	X-Clade	2 (2.6)	2 (4.8)	0 (0.0)
West African 1 (L5)	MAF-1	-	-	-
West African 2 (L6)	MAF-2	10 (12.8)	5 (11.9)	5 (13.9)
Animal lineage	M. bovis	1 (1.3)	0 (0.0)	1 (2.8)
Unclassifiable	Families 34	1 (1.3)	0 (0.0)	1 (2.8)

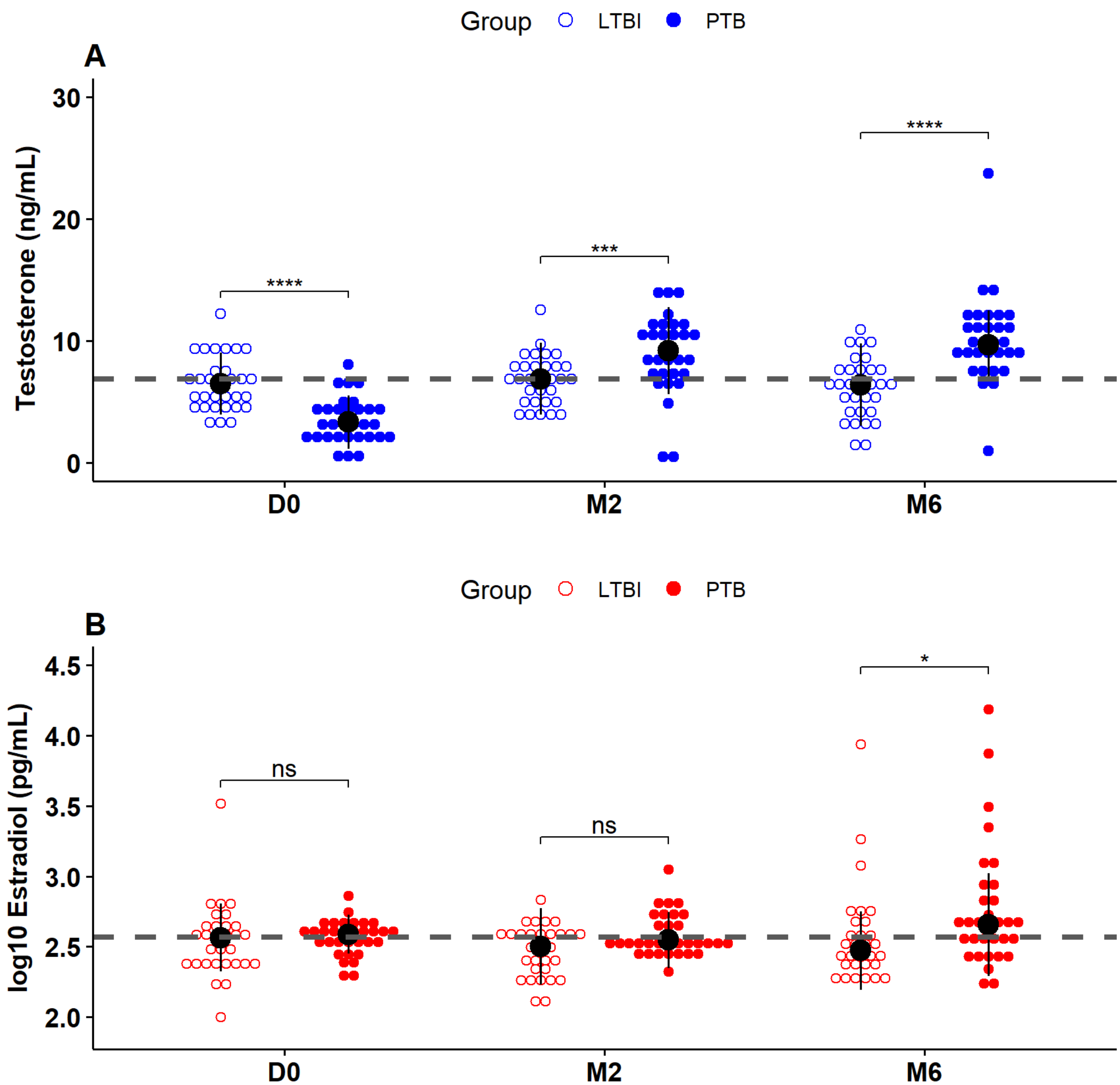

Supplement 2: Comparison of gonadal steroid concentrations between PTB and LTBI according to sex.

These figures compare testosterone (**A**) and estradiol (**B**) concentrations at three time points between males (blue) and females (red) PTB and LTBI cases. Individual values for each participant and median ± interquartile range (IQR) are presented. Wilcoxon rank-sum test has been used for group comparisons. Dashed lines show median testosterone and estradiol concentrations of sex and age-matched healthy controls, ns means non-significant, *p<0.05; **p<0.001; ***p<0.0001.

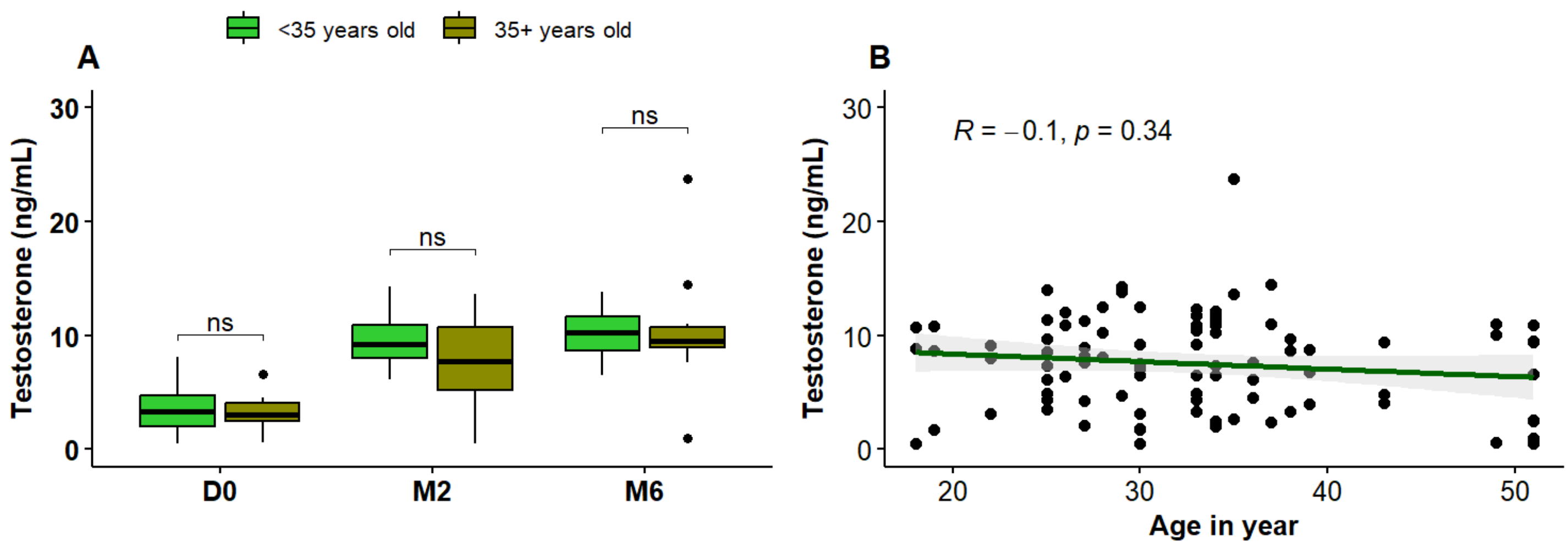

Supplement 3: Relationship between testosterone concentrations and age

Study participants were categorized in two groups: less than 35 years old (green) and 35 years old and more (brown). Panel **A** shows comparison of testosterone concentration between age groups at each time point using Wilcoxon rank-sum test. Panel **B** indicates a correlation analysis (spearman test) between participants' age and testosterone concentrations. Linear correlation line is indicated in green, and the 95% confidence interval of the correlation line is shown in gray. ns means non-significant and R represents the correlation coefficient.

.

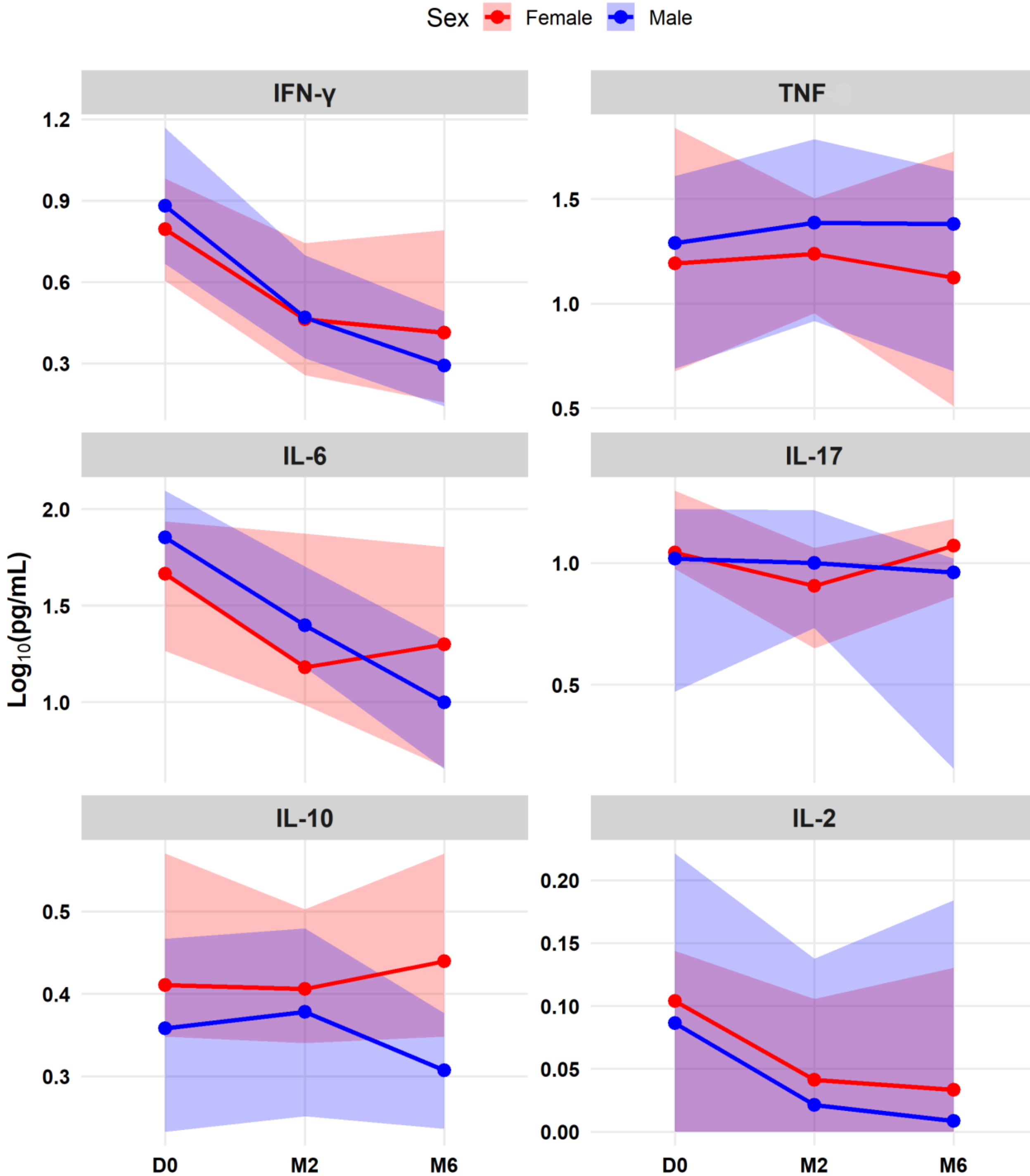

Supplement 4: Sex-specific temporal changes in cytokine levels during PTB treatment

Each panel represents longitudinal variation in cytokine levels. The name of the targeted cytokine is indicated on the top of the panel within a gray box. X axis shows cytokine levels in Log_{10}. Y axis represents the three study visits at D0, M2, and M6 during TB treatment. The thick line indicates median cytokine levels in blue for males and red for females. Shaded areas in light blue and light red represent the interquartile range (IQR) of cytokine levels in Log_{10}, respectively for males and females.
